# Contribution of early nutrition on the development of malnutrition and allergic diseases in the first year of life: a study protocol for the Mother and Infant Cohort Study (MICOS)

**DOI:** 10.1186/s12887-018-1219-3

**Published:** 2018-07-18

**Authors:** Fui Chee Woon, Yit Siew Chin, Intan Hakimah Ismail, Yoke Mun Chan, Marijka Batterham, Amir Hamzah Abdul Latiff, Wan Ying Gan, Geeta Appannah

**Affiliations:** 1 Department of Nutrition and Dietetics, Faculty of Medicine and Health Sciences, Universiti Putra Malaysia, 43400 UPM Serdang, Selangor Malaysia; 2 Department of Paediatrics, Faculty of Medicine and Health Sciences, Universiti Putra Malaysia, 43400 UPM Serdang, Selangor Malaysia; 3 Malaysian Research Institute on Ageing, Universiti Putra Malaysia, 43400 UPM Serdang, Selangor Malaysia; 4 National Institute for Applied Statistics Research Australia, University of Wollongong, Northfields Ave, Wollongong, NSW 2522 Australia; 5 Allergy & Immunology Centre, Pantai Hospital Kuala Lumpur, 59100 Kuala Lumpur, Malaysia

**Keywords:** MICOS, Infant, Early nutrition, Allergic diseases, Malnutrition

## Abstract

**Background:**

Nutrition and environmental factors are essential for the education of the neonatal immune system. Epidemiological evidence has shown that malnutrition and allergic diseases that occur during early childhood share similar protective and risk factors. This paper describes the protocol of the Mother and Infant Cohort Study (MICOS), which aims to determine the contribution of early nutrition to the development of malnutrition and allergic diseases in infants’ first year of life.

**Methods:**

MICOS is a prospective cohort study conducted at selected government health clinics in two states, namely Selangor and Wilayah Persekutuan Kuala Lumpur, Malaysia. Women in their third trimester of pregnancy are recruited into the study and their infants will be followed-up at 3, 6, and 12 months of age. Information on prenatal factors including socio-demographic characteristics, obstetric history, pre-pregnancy body mass index, gestational weight gain, smoking, family history of allergic diseases, maternal dietary intake and sunlight exposure during pregnancy are obtained through face-to-face interviews. Postnatal factors including dietary intake, sun exposure, and anthropometric measurements of the mothers, as well as feeding practices, dietary intake, anthropometric measurements, and development of allergic diseases of the infants are assessed at each follow-up. Blood samples are collected from the mothers in the third trimester to determine 25-hydroxyvitamin D levels as well as from the infants at age 12 months to determine atopic sensitisation.

**Discussion:**

The concept of developmental origins of health and disease (DOHaD) which emphasises on the role of early life environments in shaping future health and disease susceptibility in adulthood has gained a huge interest in recent years. The DOHaD paradigm has influenced many fields of research including malnutrition and allergic diseases. While findings from the developed countries remain controversial, such studies are scarce in developing countries including Malaysia. The present study will determine the cause and effect relationship between early nutrition and the development of malnutrition and allergic diseases in infants’ first year of life.

## Background

Inadequate intake of energy and nutrients may lead to malnutrition in the form of muscle wasting, stunted growth, and being underweight while excessive intake may lead to being overweight and obese [1]. Both forms of malnutrition occur among Malaysians. According to the National Health and Morbidity Survey (NHMS) 2015 Malaysia, approximately 17.7% of children below five years of age were stunted, 12.4% were underweight, 8.0% were wasted, and 7.6% were overweight [2]. Childhood malnutrition is linked to a high risk of mortality, lower levels of cognitive development, an increased susceptibility to childhood infectious diseases and lower levels of labor productivity in adulthood [3–7].

Allergy is an abnormal over-reaction or hypersensitivity reaction of the body caused by specific immunologic mechanisms which occur after an exposure to substances that are normally harmless to the human body [8]. Food allergy and eczema are the first manifestations of allergy, which usually appear during the first two years of life. Although many children outgrow their allergies, some still continue to have them. Additionally, some allergic disorders can change and progress to asthma and allergic rhinitis in later childhood. This phenomenon is known as the “atopic march” [9, 10]. The International Study of Asthma and Allergies in Childhood (ISAAC) reported that 12.6% of children (6–7 years old) in Malaysia have eczema, 5.8% have asthma, and 4.8% have allergic rhinitis [11]. Childhood allergies could lead to inappropriate diet elimination when parents are incorrectly advised and thus malnutrition, which will affect the quality of life of the patients as well as their families [12–14].

Malnutrition and allergic diseases are growing public health problems worldwide and are common diseases encountered during the first two years of life [10, 15]. As recent research demonstrated that nutrition is an essential prerequisite for the functionality of the immune system, both malnutrition and allergic diseases during childhood may have negative health consequences that persist into adulthood [10, 16]. Previous studies showed a significant association between allergic diseases and malnutrition [12, 17–22]. For example, food allergies can affect the growth and nutritional status of children with eczema. Therefore, there is a need to understand the role of early nutrition in preventing the first manifestation or progression of malnutrition and allergic diseases.

There is a growing body of evidence from research demonstrating that intrauterine exposures and early postnatal environment play a crucial role in determining the health and risk of disease later in life [23–25]. In addition, evidence from research revealed that early nutrition and lifestyle factors have long-lasting programming effects on the risk of later developing associated non-communicable diseases. Insults or stimuli that occur during the critical period, from pregnancy to early infancy, can trigger adaptations that lead to permanent changes in the structure and function of an organism, known as “programming” [26]. Early nutrition has been identified as one of the most important key players in programming; and thus, the right nutrition during the critical period is crucial to ensure proper growth and good health [25, 27].

The concept of early life nutrition refers to the maternal diet during pregnancy and lactation, as well as child feeding practices (breastfeeding and complementary feeding) [28]. Maternal nutrient requirements during pregnancy and lactation are increased in order to support fetal growth and production of breast milk [29]. During pregnancy, the supply of nutrients to the fetus is dependent on what mothers eat and the effectiveness of the placenta in transporting these nutrients to the fetus. A fetus may become undernourished when the nutrient supply does not meet its demand, thus resulting in fetal growth restriction, which is a major determinant of stunted linear growth and subsequent obesity in childhood [30]. On the other hand, maternal diet during lactation could influence her breast milk composition. Breastfeeding may protect infants against rapid weight gain and later obesity, which is possibly attributed to the bioactive components in breast milk that regulate an infant’s appetite, metabolism, weight gain, and adiposity [31].

There are certain food items in a mother’s diet during pregnancy and lactation such as fish and shellfish, peanut, and milk, which are potential food allergens, and could influence the risk of allergy among infants through in-utero allergen exposure transplacentally or transamniotically [32–34]. In-utero allergen exposure could influence the fetal immune response to shift towards development of tolerance or development of an allergic disease [34, 35]. Maternal dietary allergen exposure during lactation could influence the risk of allergy among infants through food allergens that are passed through human milk [36] which might promote tolerance in a newborn and subsequently reduce the risk of allergic diseases [18, 37]. Breast milk consists of an abundance of immunomodulatory components such as IgA, cytokines, chemokines, growth factors, and essential fatty acids which are essential to promote the development of the infant immune system [38–40]. A shorter duration of breastfeeding has been shown to be associated with an increased risk of asthma and allergic diseases in infants [41, 42]. Meanwhile, early introduction to allergenic food might decrease the risk of allergic diseases by promoting tolerance in infants [43, 44]. Apart from dietary allergen exposure, maternal intake of specific nutrients such as vitamin D and polyunsaturated fatty acids (PUFA) during pregnancy may also affect the risk of development of allergic diseases in offspring. Several studies from Western countries found that high maternal vitamin D and total PUFA intake during pregnancy were associated with a decreased risk of allergic diseases in children [45–48].

Although there are many prospective cohort studies on the association between early life nutrition and childhood malnutrition or allergy, the majority of these works were conducted in developed countries and some of the outcomes remain controversial [17–20, 22, 46, 49]. In addition, these studies focused on a single outcome, even though both allergy and malnutrition share a similar risk factor, which is early life nutrition. The Mother and Infant Cohort Study (MICOS) is therefore designed to determine the association between early life nutrition and the development of malnutrition and allergy in infants. The prospective cohort study design of MICOS involves an assessment of pre- and postnatal dietary exposures at multiple time points. Additionally, the environmental factors, family history, and maternal obstetric history are assessed to provide a comprehensive assessment of factors related to the development of childhood malnutrition and allergy. The prevalence of allergic diseases and malnutrition will be assessed and the scientific evidence on the cause and effect relationship between early nutrition and the development of allergic diseases and malnutrition in infants will be investigated. The aim of this paper is to describe the rationale and methodology of MICOS in addressing the need to investigate the association of early nutrition with malnutrition and allergy.

### Aim of the study

The present study aims to determine the contribution of early nutrition on the development of malnutrition and allergic diseases in infants at 12 months of age. The specific research questions to be answered by this study are as follows:

- What is the incidence of malnutrition in infants at 12 months of age?
- What is the incidence of allergic diseases in infants at 12 months of age?
- Is early nutrition associated with the development of malnutrition and allergic diseases in infants at 12 months of age?
- Is there any association between development of allergic diseases and malnutrition in infants at 12 months of age?

## Methods/design

### Study design and setting

**Fig. 1:**
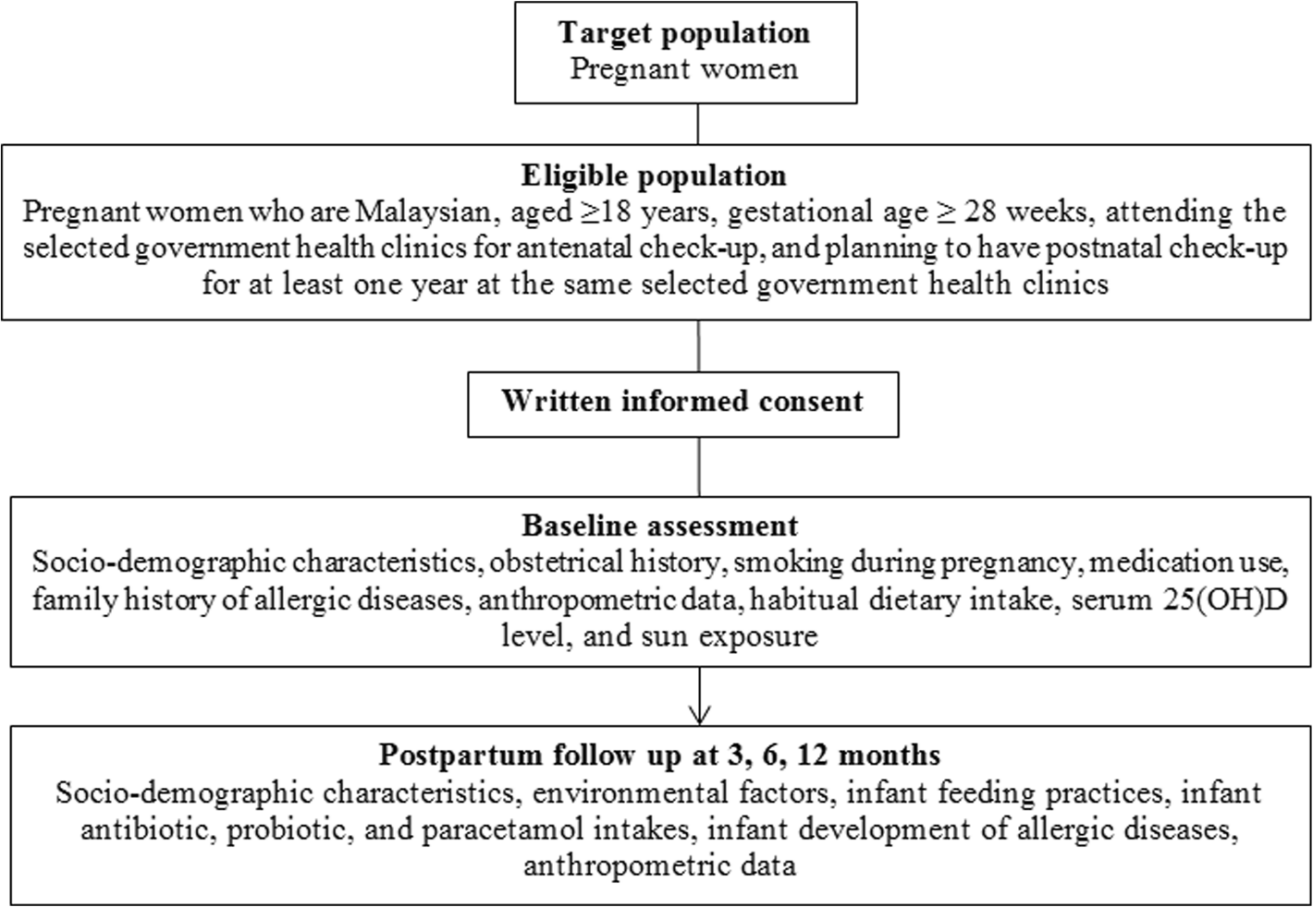
Flow chart of the cohort study MICOS

MICOS is a prospective cohort study involving pregnant women in their third trimester of pregnancy (≥ 28 weeks of gestation) who are attending six randomly selected Maternal and Child Health clinics in the state of Selangor and the city of Kuala Lumpur, Malaysia. The Maternal and Child Health (MCH) clinics are the primary source providing antenatal and postnatal care to pregnant women. In the present study, pregnant women are enrolled at ≥28 weeks of gestation and are followed-up prospectively at 3, 6, and 12 months postpartum together with their infants (Fig. 1).

### Recruitment of respondents

The respondents are selected using a cluster sampling method. A list of government health clinics in Selangor and Kuala Lumpur was obtained from the Selangor and Kuala Lumpur Health Departments. Six health clinics that met the inclusion criteria (government-funded and have a MCH clinic) were randomly selected. Pregnant women who are Malaysian, aged 18 years and above, gestational age ≥ 28 weeks, attending the selected government health clinics for antenatal check-up, and are planning to have postnatal check-up for at least one year at the same selected government health clinics are eligible to participate in this study. Women will be excluded if they are diagnosed with an immune deficiency, have a multiple pregnancy, have a preterm delivery before 37 weeks, or if their baby is born with congenital abnormalities. The objective of the study and the study procedure will be explained to the potential respondents at the clinic waiting area whilst they are waiting their turn for the antenatal check-up. Written informed consent for the respondents and their baby are obtained from the respondents who agree to participate in the study.

### Sample size calculation

Sample size was calculated using the formula for cohort study [50] with 95% power and 5% significance level. A total of 371 pregnant women is required for the study. Taking into account for a design effect of 1.119 [51] and a possible attrition rate of 28.5% [52], the sample size is increased to 533 pregnant women.

### Data collection

Recruitment of respondents began in November 2016 and is currently on-going. Respondents are followed over time and the details of the variables assessed at each assessment point in this study are shown in Table 1.

**Table 1:** Summary of data collection and timeline (Under section: Data collection - page 10)

Variables	Prenatal	Postnatal		
	3rd trimester	3 months	6 months	12 months
Mothers				
Age	√			
Ethnicity	√			
Educational level	√			
Occupation	√			
Monthly household income	√			
Obstetric history	√			
Pre-pregnancy body weight and height	√			
Body weight during pregnancy	√			
Body weight after delivery		√	√	√
Smoking during pregnancy	√			
Habitual dietary intake	√	√	√	√
Sun exposure	√	√	√	√
Serum 25(OH)D level	√			
Infants				
Sex		√		
Mode of delivery		√		
Body weight, length, head circumferences		√	√	√
Family history of allergic diseases	√			
Pet ownership		√	√	√
Day care attendance		√	√	√
Number of siblings		√		
Environmental tobacco smoke exposure		√	√	√
Infant feeding practices		√	√	√
Antibiotic, probiotic, and paracetamol intakes		√	√	√
Development of allergic diseases		√	√	√
Atopic sensitization				√

### Instrumentations

#### Maternal questionnaires

At the first encounter, information is gathered from women who are in their third trimester of pregnancy by a face-to-face interview. The information gathered includes socio-demographic characteristics (including age, ethnicity, marital status, educational level, occupation, and monthly household income), obstetrical history, smoking during pregnancy, medication use, and family history of allergic diseases. Body weight and height of the pregnant women before and during pregnancy are extracted from their medical records, while body weight after delivery is measured at 3, 6, and 12 months. The measurements are recorded to the nearest 0.1 kg for weight and 0.1 cm for length, respectively. Pre-pregnancy Body Mass Index (BMI) is calculated by the weight in kilograms divided by the height in meters squared (kg/m2). Pre-pregnancy body weight status is classified into four categories based on the Institute of Medicine (IOM) Classification [53]. Total gestational weight gain (GWG) is calculated as the difference between the final recorded body weight at the last prenatal visit and the pre-pregnancy weight recorded at the first prenatal visit in the selected health clinics. The second and third trimesters mean weekly weight gain is estimated through the difference between the first and last weight recorded in the trimester divided by the number of weeks between the two observations. The maternal GWG is then categorised as inadequate, adequate, or excessive compared to the IOM [53] recommended weight gain based on their pre-pregnancy BMI group. Postpartum weight retention is calculated as the difference between the measured weight at 3, 6, and 12 months postpartum and pre-pregnancy weight, respectively.

#### Maternal habitual dietary intake

Maternal habitual dietary intake at the third trimester of pregnancy is assessed using a semi-quantitative food frequency questionnaire (FFQ), adapted from the Malaysian Adult Nutrition Survey (MANS) [54] and vitamin D FFQ [55]. Mothers are followed-up prospectively at 3, 6, and 12 months postpartum through face-to-face interviews. The serving size of the food consumed is estimated by using household measurements. The amount of food intake per day is calculated according to this formula: frequency of intake per day x serving size x total number of servings x weight of food in one serving [56]. Data obtained will then be entered into the Nutritionist Pro™ Diet Analysis software to obtain the energy and nutrient intake of the women.

### Maternal vitamin D status

A peripheral venous blood sample (2 ml) is obtained from the women during their 3rd trimester of pregnancy by the nurses via venepuncture at the antecubital area to assess for vitamin D status. The ADVIA Centaur Vitamin D Total assay is used to determine maternal serum 25 hydroxy-vitamin D (25(OH)D) level. Maternal serum 25(OH)D level is then classified into vitamin D deficiency (< 30 nmol/L), vitamin D insufficiency (30–< 50 nmol/L) or vitamin D sufficient (≥50 nmol/L) [57].

### Maternal sun exposure

Maternal exposure to direct sunlight during the third trimester of pregnancy is determined using a Seven-day Sun Exposure Record [58] and followed-up prospectively at 3, 6, and 12 months postpartum. Women are required to record the time they spent outdoors, type of clothing worn, sunscreen use, and the nature of outdoor activities during the previous week from 7 am to 7 pm. Body surface area (BSA) exposed is estimated by referring to the guidelines of clothing key [58]. Sun exposure index (SEI) is calculated by multiplying the amount of time spent outdoors with BSA exposed [58]. A higher SEI indicates a higher exposure to sunlight.

### Infant questionnaires

Infant’s sex and mode of delivery are extracted from their medical records during the follow up visit of the infants at 3 months. Environmental factors including pet ownership, daycare attendance, number of siblings, and environmental tobacco smoke exposure among the infants are obtained from their mothers through face-to-face interviews using The International Study of Asthma and Allergies in Childhood Questionnaires (ISAAC) Phase III Environmental Questionnaire [59] at 3, 6 and 12 months postpartum. Infant’s weight, recumbent length, and head circumference data from birth to 12 months are extracted from their medical records. The anthropometric data at each age month is then converted to z-scores (length-for-age z-scores (LAZ), weight-for-age z-scores (WAZ), weight-for-length z-scores (WLZ), BMI-for-age z-scores (BMIZ), and head circumference z-scores (HCZ)) by using the WHO Reference 2007 SPSS macro package [60]. Infants nutritional status is defined as stunting (LAZ < -2SD), underweight (WAZ < -2SD), wasting (WLZ < -2SD), overweight (BMIZ > + 1SD), obese (BMIZ > + 2SD), and microcephaly (HCZ < -2SD) respectively, based on the WHO Child Growth Standards [60].

### Infant feeding practices

Mothers are interviewed for infant feeding practices at 3, 6, and 12 months postpartum using the Infant and Young Child Feeding Questionnaire adapted from the Malaysian Third National Health and Morbidity Survey (NHMS III) [61] and are based on the indicators for infant and young child feeding (IYCF) suggested by WHO [62]. The seven core indicators include early initiation of breastfeeding, exclusive breastfeeding, continued breastfeeding, introduction of solid, semi-solid or soft foods, minimum dietary diversity, minimum meal frequency, and minimum acceptable diet, while the seven optional indicators include children never breastfed, continued breastfeeding, age-appropriate breastfeeding, predominant breastfeeding, duration of breastfeeding, bottle feeding, and milk feeding frequency for non-breastfed children.

### Infant antibiotic, probiotic, and paracetamol intakes

Antibiotic, probiotic, and paracetamol intake of the infants at 3, 6, and 12 months are assessed by asking the mother: “*Has your child ever consumed any antibiotic, probiotic, or paracetamol in the past three months?*” and “*If YES, how often in the past three months did your child consume it and how much did your child consume each time?”*

### Infant development of allergic diseases

#### Eczema

Mothers are interviewed for the presence of eczema in infants at 3, 6, and 12 months based on five questions of the UK Working Party’s Diagnostic Criteria for Atopic Dermatitis [63] with response options “yes” or “no”. Eczema in infants is identified by the presence of an itchy skin condition plus two or more of the following; (i) history of involvement of skin creases such as folds of elbows, behind the knees, fronts of ankles, cheeks, or around the neck; (ii) a history of atopic disease in a first-degree relative; (iii) a history of a general dry skin; and (iv) visible flexural eczema.

#### Food allergy

Food allergy in infants at 3, 6, and 12 months are assessed by asking the mothers: “Has your child ever had a skin rash and sickness within two hours of eating some food?” and “Did these symptoms repeat each time the same food was consumed?” [64]. If positive answers are given to both of these questions, the mothers are required to select the type of food their children consumed that resulted in those symptoms. Options to select from include egg, peanut, tree nut, milk, shellfish, fish, wheat, and soy.

#### Asthma

The Asthma Predictive Index (API) [65] is used to determine the likelihood of infants who may develop asthma at 3, 6, and 12 months. A ‘positive’ API involves the presence of recurrent episodes of wheezing (more than three episodes per year) and one of two major criteria: (1) Asthma in a parent or (2) Eczema in infant; or two minor criteria: (1) Allergic rhinitis in infant and (2) Wheezing apart from colds in infant.

#### Rhinitis

Rhinitis in infants at 3, 6, and 12 months is assessed by the ISAAC questionnaire [66]. An infant is labelled to have rhinitis if the mothers report that the infant had a runny nose or sneezing episodes with no evidence of cold or flu.

### Infant atopic sensitization

Peripheral venous blood samples are obtained from the infants via venepuncture at age 12 months. Approximately 1–2 mL of blood is collected by the medical assistants into 5-ml plain tubes. Serum samples are analyzed by using the OPTIGEN Allergen Specific Immunoglobulin E (IgE) Assay (Hitachi Chemical Diagnostics Inc., Japan) which enables the simultaneous determination of the infants’ total IgE and specific IgE levels to a total of 35 food and inhalant allergens (egg yolk, egg white, soybean, peanut, milk, clam, crab, shrimp, cod fish, tuna, salmon, rice, wheat, banana, orange, sesame seed, chocolate, chicken, beef, mucor, timothy grass, bermuda glass, *Alternaria*, *Aspergillus*, *Candida*, *Cladosporium*, *Penicillium*, dog dander, cat dander, cockroach mix, housedust, *Mite Farinae*, *Mie Pteronyssinus*, *Blomis Tropicalis*, and latex). The results obtained from the test in net luminescence units (LU), are classified into class 0 (0–26 LU), class 1 (27–65 IU), class 2 (66–142 LU), class 3 (143–242 LU) and class 4 (> 243 LU) using the Chemiluminescent Assay (CLA) Class Allergy Scoring System (Hitachi Chemical Diagnostics Inc., Japan). Class ≥1 is interpreted as positive, indicating that the infants are sensitised to a specific food or aero-allergens.

### Data analysis

The IBM SPSS Statistics 24 software (SPSS Inc., Chicago, IL, USA) will be used to analyse the data. Descriptive statistics and univariate analysis will be performed to describe the data. Hierarchical linear regression analysis with confounders are forcibly entered to examine the association between various exposure variables and the longitudinal outcomes. Data will be presented as relative risk (RR) with 95% confidence interval. Kaplan-Meier test and Cox regression analysis will be performed to analyse the time-to-event data and hazard ratios (HR) with a 95% confidence interval will be reported.

## Discussion

About 60% of allergies appear during the first year of life [10]. The “hygiene hypothesis” originally proposed by Strachan [67] suggests that environmental influences such as decreased or absence of microbial exposures in early life have an adverse effect on the development of the immune system, which may lead to the development of allergic diseases. The concept of early environmental influences on later disease also draws on the increasing interest in fetal programming, known as the “Barker’s hypothesis” [23]. Barker suggested that nutritional conditions during fetal life can influence the metabolism and occurrence of disease during adult life. Fetal undernutrition in middle to late gestation can affect fetal growth, which may contribute to an increased risk of non-communicable diseases such as coronary heart disease in later life. Barker’s hypothesis was then further extended to the developmental origins of health and disease (DOHaD) which emphasises the role of both the pre- and postnatal nutritional environment in determining adult diseases [24]. These three hypotheses suggests that early life nutritional environment can have lifetime consequences on later health. Hence, understanding the contribution of early nutrition, from pregnancy to early infancy is important to prevent the first manifestation of allergy or its progression, as well as early childhood malnutrition, which in turn lowers the risk of diseases in later life.

The prospective cohort study design of MICOS will generate a better understanding on the cause-effect relationship between early life nutrition and development of childhood malnutrition and allergy. In Malaysia, studies that examined the concept of early nutritional programming using a cohort study design are scarce. The USM Pregnancy Cohort Study was the first cohort study conducted in the state of Kelantan, Malaysia that linked maternal dietary exposures during pregnancy with birth outcomes in infants [68]. Another cohort study is being conducted in the state of Negeri Sembilan, Malaysia to determine early nutrition, growth and cognitive development of infants from birth to 2 years of age and is currently on-going [69]. Hence, the results of this study will fill the knowledge gap in this region by providing evidence for the role of early nutrition on growth and allergy development. In addition, the IgE blood test used in MICOS will help in identifying the prevalence of allergen sensitisation among infants in Malaysia. The incidence of allergic diseases and malnutrition that will be reported in the present study can enlighten the health professionals, policy makers as well as the public on the importance of early diagnosis of allergic diseases and malnutrition among infants. Through this study, we expect to contribute new knowledge and evidence of the association between early nutrition, childhood malnutrition and allergy which may be useful in helping health professionals and policy makers to develop dietary practice guidelines for pregnant women and infants to optimise the early life environment to ensure the health of future generations.

## Abbreviations

25(OH)D: 25 hydroxy-vitamin D
BMI: Body mass index
BSA: Body surface area
CLA: Chemiluminescent assay
FFQ: Food frequency questionnaire
GWG: Gestational weight gain
IgE: Immunoglobulin E
IOM: Institute of Medicine
ISAAC: International Study of Asthma and Allergies in Childhood
IYCF: Indicators for infant and young child feeding
JKEUPM: Ethics Committee for Research Involving Human Subjects, Universiti Putra Malaysia
LU: Net luminescence units
MANS: Malaysian Adult Nutrition Survey
MICOS: Mother and Infant Cohort Study
MREC: Medical Research and Ethics Committee
NHMS: National Health and Morbidity Survey
RNI: Recommended Nutrient Intakes for Malaysians
SEI: Sun exposure index
UK: United Kingdom
WHO: World Health Organization
